# Bioinformatics and Expression Analysis of CHI Gene Family in Sweet Potato

**DOI:** 10.3390/plants14050752

**Published:** 2025-03-01

**Authors:** Yaqin Wu, Xiaojie Jin, Lianjun Wang, Chong Wang, Jian Lei, Shasha Chai, Wenying Zhang, Xinsun Yang, Rui Pan

**Affiliations:** 1 College of Agriculture, Yangtze University, Jingzhou 434025, China; 17300720603@163.com (Y.W.); wyzhang@yangtzeu.edu.cn (W.Z.); 2 Institute of Food Crops, Hubei Academy of Agricultural Sciences, Wuhan 430064, China; xiaojiejin@hbaas.com (X.J.); wanglianjun@hbaas.com (L.W.); leijian2006@hbaas.com (J.L.); chaishasha2008@hbaas.com (S.C.); 3 Institute of Crops, Jiangxi Academy of Agricultural Sciences, Nanchang 330200, China; wangchong19409@163.com; 4 Hubei Potato and Taro Industry Technology Research Institute Co., Ltd., Huanggang 438400, China

**Keywords:** sweet potato, chalcone isomerase, abiotic stress, bioinformatics analysis, expression analysis

## Abstract

Chalcone isomerase (CHI) is not only an enzyme related to flavonoid biosynthesis, but also one of the key enzymes in the flavonoid metabolic pathway. In this study, members of the CHI gene family were identified in the whole genome of sweet potato. Bioinformatics methods were used to analyze the physical and chemical properties, systematic evolution, conserved domain, chromosome location, cis-acting elements of the promoter, and so on, of CHI gene family members. In addition, the tissue site-specific expression of CHI gene family members and their expression patterns under three kinds of abiotic stress were analyzed. The results showed that five members of IbCHI gene family were identified in sweet potato, which were unevenly distributed on four chromosomes. The protein secondary structure and tertiary structure were consistent, and there was a conservative domain related to chalcone isomerase. The prediction of subcellular localization showed that it was mainly located in cytoplasm and chloroplast. Systematic evolution showed that the members of sweet potato CHI gene family could be divided into Type I-IV, and the Type I gene *IbCHI1* showed CHI catalytic activity in transgenic callus. The collinearity gene pairs were identified between sweet potato and allied species. Its promoter contains light response elements, hormone response elements, and stress response elements. The results of real-time fluorescence quantitative PCR (qRT-PCR) analysis showed that the expression of the IbCHI gene was tissue-specific and that the catalytic genes *IbCHI1* and *IbCHI5* serve as primary responders to abiotic stress, while the non-catalytic members *IbCHI3* and *IbCHI4* may fine-tune metabolic flux or participate in low-temperature, salt, and drought stress signaling. This study can provide a theoretical basis for a follow-up functional genomics study of the chalcone isomerase gene family in sweet potato.

## 1. Introduction

Sweet potato (*Ipomoea batatas* (L.) Lam.) belongs to the *Convolvulaceae* family and the *Ipomoea* genus and is a perennial dicotyledonous plant that prefers light and adapts to short day conditions [1,2]. Sweet potato is an important cereal crop that can be used for food processing, feed processing, and energy purposes. Sweet potatoes are widely planted in over 100 countries worldwide, only ranking below rice, wheat, potatoes, corn, and cassava, and are an indispensable crop resource [3]. China occupies a dominant position in global sweet potato production, with planting area accounting for about 30% of the total global sweet potato area and production accounting for about 58% of the total global production [4]. Sweet potatoes are rich in dietary fiber, sugar, minerals, vitamins, and various essential nutrients for human health, and are known as a highly nutritious and preferred food [5].

Secondary metabolites are widely involved in the growth and development of plants and their response to adversity. According to their different chemical structures and biological functions, plant secondary metabolites can be divided into multiple categories, including phenylpropanoids, quinones, flavonoids, tannins, terpenes, steroids and their glycosides, alkaloids, and seven other major categories [6]. Chalcone isomerase (CHI), as a key rate-limiting enzyme in the synthesis pathway of flavonoids, has a relatively small molecular weight and plays an indispensable role in plant growth and development, pigment accumulation, defense reactions, and signal transduction [7]. The content of flavonoids in plants is influenced by the expression level of *CHI* genes. Flavonoids, as one of the most important secondary metabolites, can enhance plant stress resistance by scavenging reactive oxygen species under abiotic stresses (drought, salinity, extreme temperatures, etc.) [8]. In 1987, the coding gene for chalcone isomerase was first cloned from French peas using antibody technology [9]. With the continuous deepening of research, more and more *CHI* genes have been identified and reported in plants, including *Arabidopsis thaliana* [10], rice (*Oryza sativa* L.) [11,12], maize (*Zea mays* L.) [13], barley (*Hordeum vulgare* L.) [12], peanuts (*Arachis hypogaea* L.) [14], kiwifruit (*Actinidia chrysantha* Planch) [15], *Ginkgo biloba* L. [16], soybean (*Glycine max*) [17], alfalfa (*Medicago sativa*) [18], pea (*Pisum sativum*) [19], and other species. Research has found that under salt stress, the expression level of the CHI gene in *Populus tomentosa* increases, enhancing its salt tolerance [20]. The expression of the *HvCHI* gene in barley sharply decreases under drought and low-temperature stress [21]. The *CHI* gene family has been identified and analyzed in species such as soybean [22], purple clover [23], and golden flower tea [24]. In addition, 11 *CHI* family genes were identified from the dragon blood tree, and it was found that *DcCHI1* and *DcCHI4* play important roles in flavonoid synthesis [25].

There are few reports on the study of chalcone isomerase in sweet potatoes. Previous studies have shown that flavonoids, mainly composed of CHI genes, play an important role in responding to abiotic stress [26,27], but research on the sweet potato *CHI* gene family is still extremely limited. This study identified the *CHI* gene family through the sweet potato genome and analyzed the protein physicochemical properties, gene structure, chromosome localization, evolutionary development, and expression patterns of the *CHI* gene family using a bioinformatics system. Using the sweet potato variety ‘EC03’ as the experimental material, the tissue-specific expression of *CHI* gene family members and their expression patterns under three types of abiotic stress were analyzed. This study will provide a theoretical basis for the functional analysis of the chalcone isomerase gene family in sweet potato and provide genetic resources for sweet potato breeding and variety improvement.

## 2. Results

### 2.1. Subsection Identification of Sweet Potato CHI Gene Family Members

**Figure 1:**
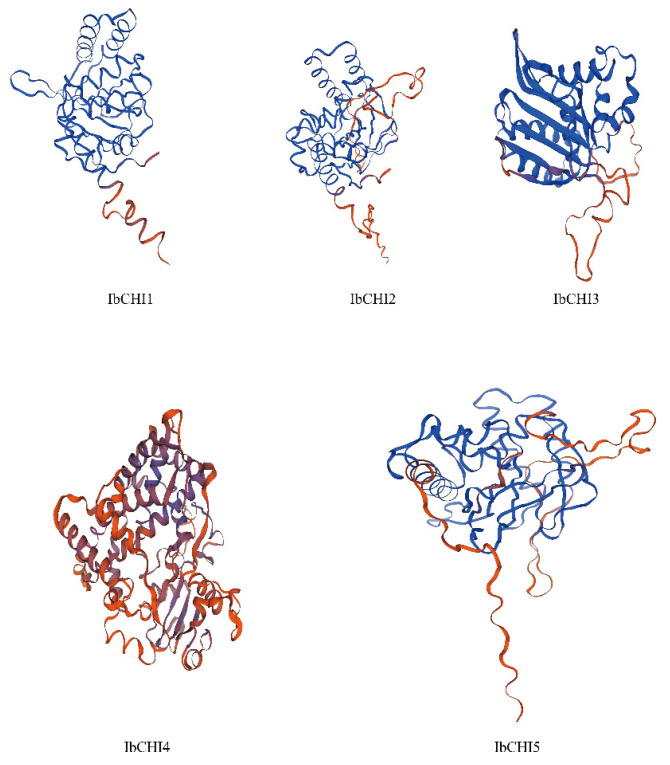
Protein tertiary structure prediction of *IbCHI*.

Five *CHI* gene family members were identified in the sweet potato genome and named *IbCHI1*-*IbCHI5* based on their distribution on chromosomes. There are significant differences in CDS length, amino acid number, and relative molecular weight among members of the sweet potato *CHI* gene family. The CDS size ranges from 732 to 2382 bp and the number of amino acids is 243–793 aa. Among them, *IbCHI4* has the longest CDS and protein amino acid length, while *IbCHI1* has the shortest. The relative molecular weight ranges from 25.66 to 88.75 kDa, with the largest being *IbCHI4* and the smallest being *IbCHI1*. The isoelectric point is 4.78–9.22. Except for *IbCHI4*, which has an isoelectric point of 9.22, the isoelectric points of all other *CHI* proteins are less than 7.00, indicating that they belong to a class of acidic proteins. The instability coefficient ranges from 35.49 to 48.49, with three members being stable proteins. The average hydrophilicity is −0.123–0.044, with three members being hydrophilic proteins (Table 1). Subcellular localization prediction shows that it is mainly located in the cytoplasm and chloroplasts (Table 2). The prediction of the secondary structure of *CHI* protein shows that the secondary structure of sweet potato *CHI* gene family proteins is composed of an α-helix structure (accounting for 27.22–38.21%), irregular coiled structure (accounting for 32.11–40.97%), and extended chain structure (18.41–25.88%). The proportion of beta angle structures (5.93–9.88%) is relatively small (Table 2). The secondary and tertiary structures of sweet potato *CHI* gene family proteins have similarities (Figure 1).

### 2.2. Chromosome Mapping Analysis of Sweet Potato CHI Gene

**Figure 2:**
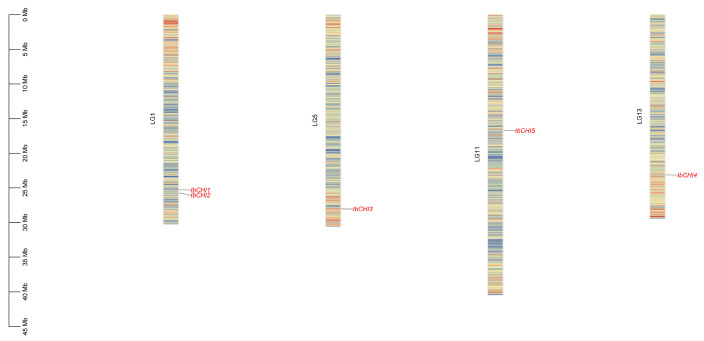
Chromosome localization of *CHI* gene family in *Ipomoea batatas*. The heatmap represents the gene distribution density on chromosomes.

According to the positional information of the sweet potato *CHI* gene family on chromosomes, five gene family members are co-located on four chromosomes (LG1, LG5, LG11, LG13) (Figure 2). There are two *CHI* gene family members, *IbCHI1* and *IbCHI2*, distributed on chromosome LG1, while one member is distributed on all other chromosomes. Except for *IbCHI5*, located at the mid-end of the chromosome, other members of the gene family are distributed at the end of the chromosome.

### 2.3. Phylogenetic Tree Analysis of Sweet Potato CHI Gene Family

**Figure 3:**
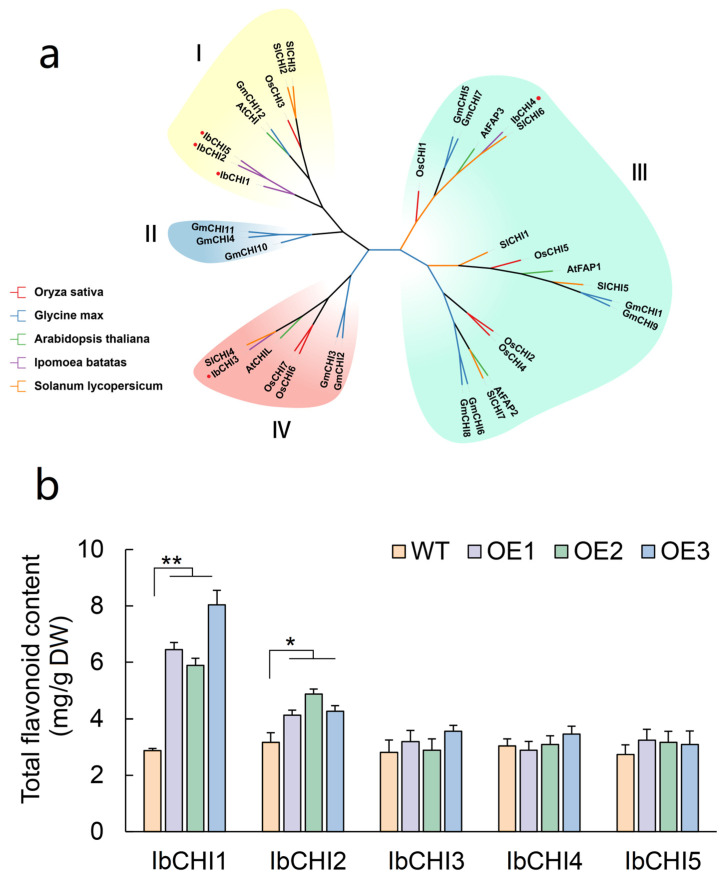
Phylogenetic and functional analysis of *CHI* gene families in *Ipomoea batatas*. (**a**) Phylogenetic analysis of CHI gene family members in *Ipomoea batatas*, *Arabidopsis thaliana*, *Glycine max*, *Oryza sativa*, and *Solanum lycopersicum.* I, II, III, IV represent type I, type II, type III and type IV CHI respectively. (**b**) Total flavonoid content quercetin equivalents in the *IbCHI1*-*IbCHI5*-overexpression calluses. * and ** stands for significance level at *p* < 0.05 and *p* < 0.01, respectively.

The evolution of the *CHI* gene family in *Ipomoea batatas*, *Arabidopsis thaliana*, *Glycine max*, *Oryza sativa*, and *Solanum lycopersicum* was analyzed by the adjacency method. The *CHI* family members were divided into four types (Type I-IV). The Type I CHI subfamily comprised one CHI gene each from *Arabidopsis thaliana* (*AtCHI*), *Oryza sativa* (*OsCHI3*), and *Glycine max* (*GmCHI12*), two CHI genes (*SlCHI2* and *SlCHI3*) from *Solanum lycopersicum*, along with three CHI genes from *Ipomoea batatas* (*IbCHI1*, *IbCHI2*, *IbCHI5*). The Type II CHI subfamily exclusively contained three CHI genes derived from *Glycine max*. Based on previous studies, Type I and Type II CHI genes exhibit authentic CHI catalytic activity [10]. This supports the hypothesis that *IbCHI1*, *IbCHI2*, and *IbCHI5* in sweet potato likely function as catalytic enzymes participating in flavonoid biosynthesis. The CHI gene family members in Type III could be further subdivided into three subclasses: FPA1, FPA2, and FPA3. Notably, *IbCHI4* from *Ipomoea batatas* clustered within the FPA3 subclass, suggesting its potential involvement in fatty acid metabolism. In contrast, the Type IV CHI subfamily gene, *IbCHI3*, may contribute to flavonoid synthesis through a CHI non-catalytic activity (Figure 3a).

CHI enzymes typically rely on conserved amino acid residues for substrate binding. In Type I/II CHIs, the conserved residues Lys 93, Ile/Leu 100, Tyr 108, Lys 111, Ala 188, and Val 189 are substituted in Type III CHIs with Met 93, Val 100, Val 108, Ala 111, Ala 188, and Ile/Leu/Phe 189, respectively. Type IV CHIs exhibit substitutions at positions Ser/Thr 104, Lys 111, Ala 188, and Val 189, replacing the Type I counterparts Lys 104, Gln 111, Met 188, and Ile/Met 189. These divergent substitutions in conserved residues likely impair substrate binding in Type III and Type IV CHI family proteins, thereby abolishing their CHI catalytic activity. Notably, three critical residues in Type I CHIs—Thr/Met 95 (hydrogen bonding site), Ser/Thr 104 (dual role in hydrogen bonding and substrate-binding cleft formation), and Gln 107 (substrate-binding cleft)—are replaced by Ile 95, Asp 104, and Glu 107 in Type II CHIs. While Type I and Type II CHIs retain catalytic activity, these structural variations account for their distinct substrate preferences and catalytic efficiencies. Compared to other species, sweet potato exhibits a single substitution (from Thr 104 to Ser 104), while all other residues remain conserved, indicating strong functional conservation of Type I CHI genes in this species (Figure S1).

To identify functional chalcone isomerase (CHI) genes in *Ipomoea batatas*, a comparative analysis of total flavonoid content was conducted in transgenic callus lines overexpressing individual CHI genes (Figure S2). Quantitative measurements revealed that *IbCHI1*-overexpressing callus accumulated 2.36-fold total flavonoid content, compared to wild-type callus (control) (*p* < 0.01), confirming its role as the principal CHI active enzyme. A moderate 0.39-fold increase (*p* < 0.05) observed in *IbCHI2*-overexpressing lines suggested residual enzymatic activity. No significant differences (*p* > 0.05 by ANOVA with Tukey’s test) were detected between lines *IbCHI3*, *IbCHI4*, or *IbCHI5*-overexpressing calluses and control, demonstrating the absence of CHI functionality in these paralogs (Figure 3b).

### 2.4. Analysis of CHI Gene Structure and Promoter Elements in Sweet Potato

**Figure 4:**
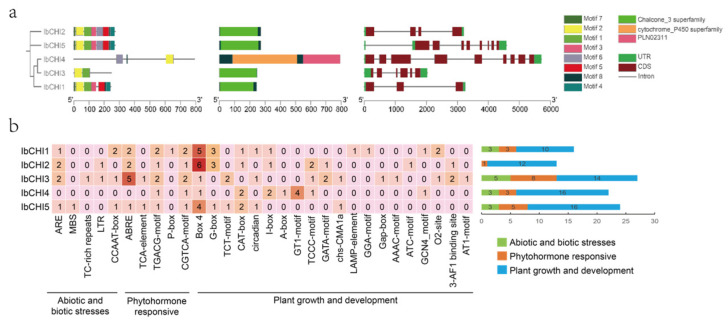
Analysis of *CHI* gene family evolution, conserved motif, protein conserved domain, gene structure, and promoter elements in sweet potato. (**a**) *CHI* gene family conserved motif, protein conserved domain, and gene structure. (**b**) Promoter element analysis. The darker the red, the greater the number.

Using MEME and NCBI-CDD websites to analyze the conserved motifs, protein conserved domains, and gene structure of sweet potato *CHI* protein. The eight conserved motifs identified from the *CHI* gene family will be named Motif 1–Motif 8, which share a common conserved motif, Motif 2. Except for *IbCHI4*, the other four members all contain Motif 1, indicating their functional similarity. Except for *IbCHI3*, the other four members all contain Motif 8, suggesting that Motif 1 and Motif 8 are important motifs determining the function of *CHI* family genes. Analysis of the conserved domains of proteins in the *CHI* family revealed that except for *IbCHI4*, all other proteins contain the Chalcone3 superfamily conserved domain. There are differences in the number of exons and introns in the sweet potato *IbCHI* gene family. *IbCHI4* has the highest number of exons and introns, with nine and eight, respectively, while *IbCHI1* has the lowest number of exons and introns, with three and two, respectively. In addition, all members of the *CHI* gene family contain untranslated regions (UTRs), and there are significant differences in the distribution and length of UTRs among members, indicating structural diversity and functional diversity among *CHI* gene family members. It is inferred that there may be functional complementarity among members (Figure 4a).

The type and distribution of cis elements within promoters of CHI gene family members were revealed in sweet potato. The cis-acting elements of CHI genes’ promoters in sweet potato were mainly divided into three categories: biological and abiotic stress, hormone response, and growth and development (Figure 4b). The results showed that the distribution of cis-acting elements related to plant growth and development was the highest in the promoters of CHI gene family of sweet potato, followed by hormone response, and the distribution of biological and abiotic stress-related cis-acting elements was the least. Notably, the count of photoresponsive element Box 4 was more in Type I CHI genes (5, 6 and 4 in *IbCHI1*, *IbCHI2*, and *IbCHI5*, respectively) than in the Type III CHI gene *IbCHI4* and Type IV CHI gene *IbCHI3* (only one in each gene), suggesting that these elements may be important factors affecting flavonoid synthesis under light exposure. The promoters of all CHI genes in sweet potato contain TGACG-motif and CGTCA-motif related to methyl jasmonate (MeJA). In addition to *IbCHI4* gene, all gene promoters contain ABRE, an ABscisic acid (ABA) response element, indicating that the CHI genes may have crosstalk with the hormone signaling pathway in sweet potato. It was also found that the promoter of CHI gene family members contains several regulatory elements such as drought response element MBS and low-temperature response element LER. These results indicated that CHI genes of sweet potato were involved in the growth and development of sweet potato and stress through multiple pathways.

### 2.5. Collinearity Analysis of Sweet Potato CHI Gene Family

**Figure 5:**
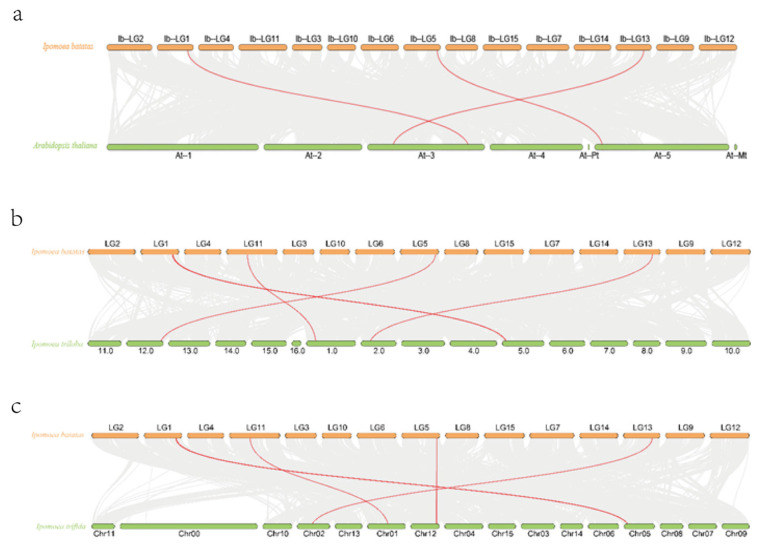
Collinearity analysis of sweet potato *CHI* gene. (**a**) Sweet potato and *Arabidopsis CHI* gene collinearity. (**b**) Sweet potato and *Ipomoea triloba CHI* gene collinearity. (**c**) Sweet potato and *Ipomoea trifida CHI* gene collinearity.

The collinearity analysis showed that there was no collinearity among the members of the sweet potato *CHI* gene family, but there was collinearity between sweet potato and the *CHI* gene families of *Arabidopsis*, *Ipomoea triloba*, and *Ipomoea trifida* (Figure 5). There are three pairs of collinear relationships between sweet potato *CHI* members and *Arabidopsis thaliana* (*IbCHI2* and *AT3G55120.1*, *IbCHI3* and *AT5G05270.1*, *IbCHI4* and *AT3G14610.1*), four pairs of collinear relationships between sweet potato *CHI* members and *Ipomoea triloba* (*IbCHI2* and *itb05g04360.t1*, *IbCHI3* and *itb12g24330.t1*, *IbCHI4* and *itb02g09710.t1*, *IbCHI5* and *itb01g08940.t2*), and four pairs of collinear relationships between sweet potato *CHI* members and *Ipomoea trifida* (*IbCHI2* and *itf05g04930.t1*, *IbCHI5* and *itf01g16540.t1*, *IbCHI3* and *itf12g23980.t1*, *IbCHI4* and *itf02g14290.t1*).

### 2.6. Analysis of Tissue Expression Characteristics of CHI Gene Family in Sweet Potato

**Figure 6:**
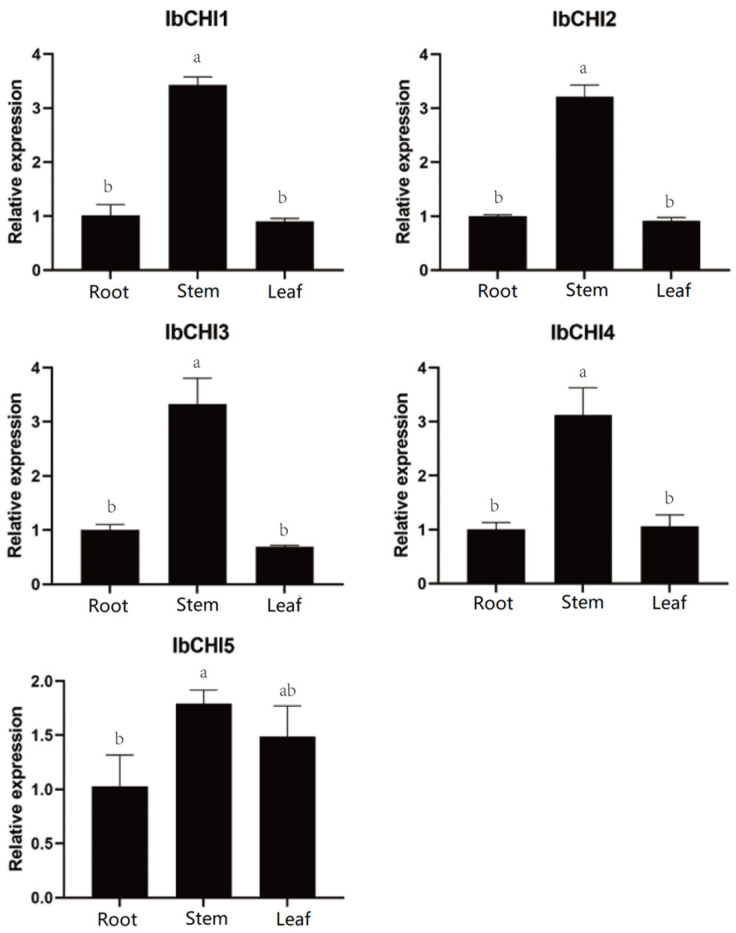
Tissue-specific expression analysis of *CHI* gene family in sweet potato. The different lowercase letters stants for the significant level at *p* < 0.05.

The expression pattern of the *IbCHI* gene in the fibrous roots, stems, and leaves of sweet potato is shown in Figure 6. The expression of the *IbCHI* gene was tissue-specific. The expression of all *IbCHI* family genes was the highest in the stem, and the expression pattern of the *IbCHI1*-*IbCHI4* gene was similar. The expression of the *IbCHI1*-*IbCHI4* gene in the stem was about three times higher than that in the root and leaf. The expression of *IbCHI1*, *IbCHI2*, and *IbCHI3* in tissues were stem > root ≈ leaf, and that of the *IbCHI5* gene was stem > leaf > root. Among tissues, *IbCHI2* showed the lowest expression levels among leaf, stem, and root, while *IbCHI5* showed the highest expression levels in leaf and root. All IbCHI gene family members except *IbCHI2* were highly expressed in the stem. The results suggest that members of the *IbCHI* gene family in sweet potatoes may exhibit functional diversity in different tissues.

### 2.7. Expression Analysis of CHI Gene Family in Sweet Potato Under Stress

#### 2.7.1. Expression Analysis of IbCHI Gene Under Low-Temperature Stress

**Figure 7:**
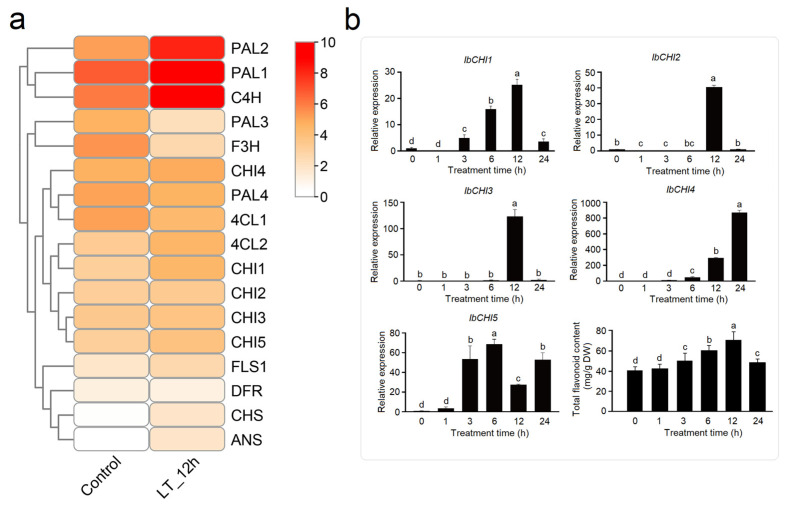
Expression analysis of flavonoid synthesis pathway genes in sweet potato in response to low-temperature stress. (**a**) The expression level in transcriptome analysis (Cold stress for 12 h); (**b**) time-dynamic expression level of CHI genes in sweet potato. “LT_12h” stands for low-temperature treatment for 12 h. The different lowercase letters stants for the significant level at *p* < 0.05.

Under cold treatment (12 h), upstream genes *PAL1*, *PAL2*, and *C4H* were markedly upregulated, though subfamily genes *PAL3* and *4CL1* were suppressed, indicating a systemic activation of the flavonoid biosynthesis pathway. Late-stage genes *CHS*, *ANS*, and *FLS1* showed cold induction, whereas *F3H* and *DFR* were downregulated or unaffected. Among the CHI genes, *CHI1* displayed the strongest cold responsiveness (2.8-fold increase), followed by *CHI5* (1.8-fold). In contrast, *CHI2*, *CHI3*, and *CHI4* exhibited minimal cold sensitivity. These results indicate isoform-specific activation of *CHI1* and *CHI5* under cold stress, suggesting their prioritized roles in redirecting metabolic flux toward stress-responsive flavonoid derivatives during cold adaptation (Figure 7a). The time-dynamic changes in CHI genes showed that the expression level of *IbCHI1* (3 h) and *IbCHI5* (3 h) was induced earlier than *IbCHI2* (12 h) and *IbCHI3* (12 h), as well as *IbCHI4*, which peaked at 24 h. Notably, the total flavonoid content was significantly increased in 3 h and peaked at 12 h, suggesting that *IbCHI1* and *IbCHI5* play roles in early cold-induced flavonoid synthesis (Figure 7b).

#### 2.7.2. Expression Analysis of IbCHI Gene Under High-Salt Stress

**Figure 8:**
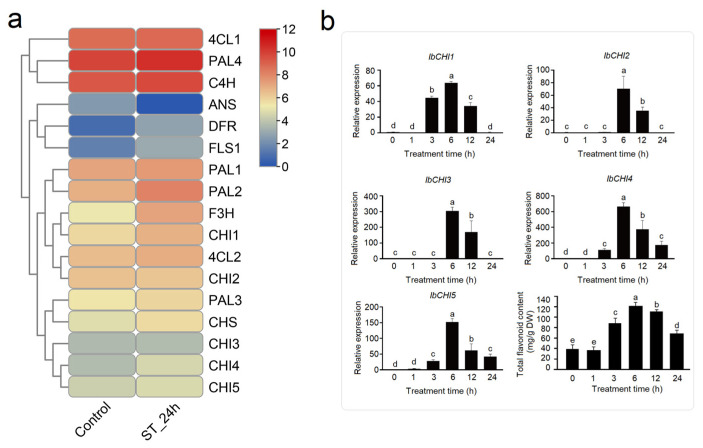
Expression analysis of flavonoid synthesis pathway genes in sweet potato in response to high-salt stress. (**a**) The expression level in transcriptome analysis (Salt stress for 24 h); (**b**) time-dynamic expression level of CHI genes in sweet potato. “ST_24h” stands for salt treatment for 12 h. The different lowercase letters stants for the significant level at *p* < 0.05.

Under salt stress (24 h), the flavonoid biosynthesis pathway demonstrated broad activation with distinct regulatory patterns. Upstream genes, including *phenylalanine ammonia lyase* (*PAL2*, *PAL4*), *cinnamate 4-hydroxylas* (*C4H*), *4-coumarate-CoA ligases* (*4CL2*), and *chalcone synthase* (*CHS*), were moderately upregulated, suggesting enhanced precursor supply. Notably, *flavanone 3-hydroxylase* (*F3H*), *dihydroflavonol 4-reductase* (*DFR*), and *flavonol synthase* (*FLS1*) exhibited pronounced induction, while *ANS* was sharply repressed, indicating a potential shift toward flavanol or flavonol production over anthocyanin synthesis. *IbCHI1* and *IbCHI4* showed strong salt-responsive upregulation (2.1 and 2.0-fold increase, respectively), *IbCHI5* was slightly increased, and *IbCHI2* and *IbCHI3* remained largely unaffected. The preferential induction of *IbCHI1* and *IbCHI4* implies their specialized roles in salt adaptation, potentially facilitating the biosynthesis of stress-protective flavonoids (Figure 8a). Time-scale changes showed that the expression levels of all *IbCHI* genes peaked at 6 h; however, *IbCHI1*, *IbCHI4*, and *IbCHI5* were induced from 3 h, and *IbCHI2* and *IbCHI3* from 6 h. The total flavonoid contents significantly increased from 3 h and peaked at 6 h (Figure 8b). *IbCHI4*, belonging to the type III subfamily, clearly lacks CHI activity, it may respond to salt stress through other pathways. The results indicated the potential CHI catalytic function of *IbCHI1* and *IbCHI5*.

#### 2.7.3. Expression Analysis of IbCHI Gene Under Drought Stress

**Figure 9:**
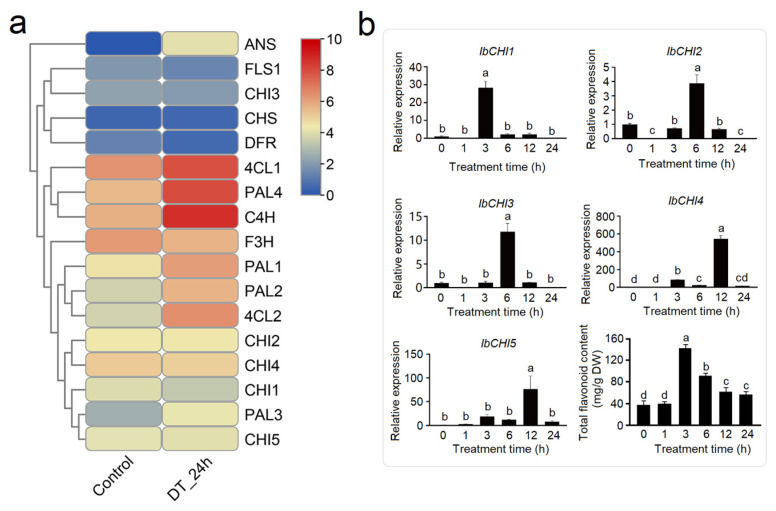
Expression analysis of flavonoid synthesis pathway genes in sweet potato in response to drought stress. (**a**) The expression level in transcriptome analysis (drought stress for 24 h); (**b**) time-dynamic expression level of CHI genes in sweet potato. “DT_24h” stands for drought treatment for 24 h. The different lowercase letters stants for the significant level at *p* < 0.05.

Under drought stress (24 h), upstream genes, including *PAL1*, *PAL2*, *PAL3*, *PAL4*, *C4H*, *4CL1*, and *4CL2*, were strongly upregulated, indicating enhanced precursor flux into the pathway. In contrast, late-stage genes exhibited mixed responses; *ANS* showed dramatic upregulation, while *F3H*, *DFR*, and *FLS1* were suppressed. *CHS* remained largely unchanged (Figure 9a). The expression levels of all *CHI* genes showed no significant changes, and temporal dynamics provided a comprehensive explanation; the expression level of *IbCHI1* peaked at 3 h, *IbCHI2* and *IbCHI3* at 6 h, *IbCHI4* and *IbCHI5* at 12 h, and then all *IbCHI* genes’ expression levels decreased to low levels at 24 h. Interestingly, the total flavonoid content peaked at 3 h and then significantly decreased with prolonged treatment time, indicating that *IbCHI1*, the only one high-expressed at 3 h, plays a crucial role in the accumulation of flavonoids in the early stages of drought (Figure 9b).

## 3. Discussion

Sweet potato is rich in a variety of ingredients that are beneficial to human health. Its unique ingredients have a variety of physiological activities such as antioxidant, liver protection, anti-tumor, anti-diabetes, antibacterial, anti-obesity and anti-aging [28]. Flavonoids play a crucial role in this process. As a key enzyme in flavonoid biosynthesis, chalcone isomerase (CHI) catalyzes the stereospecific isomerization of naringenin chalcone to (2S)-flavanone [29]. It catalyzes the stereospecificity and intramolecular isomerization of naringenin chalcone, producing the corresponding (2S)—flavanone [30]. This isomerization reaction can occur spontaneously, and with the participation of *CHI*, its turnover rate increased by 107 times [31]. *CHI* is usually divided into four types: type I, II, III and IV [10,17]. Among them, type I and type II *CHI* proteins are true *CHI* and have catalytic activity. It is worth noting that type III/IV *CHI* protein also plays an important scaffold role in flavonoid transport, type III *CHI* is a fatty acid binding protein involved in fatty acid metabolism [10], and type IV *CHI* protein can be used as an enhancer (activator) to promote the production of plant flavonoids [32,33]. Our systematic identification of five CHI family members (*IbCHI1*–*IbCHI5*) revealed significant structural and functional divergence. Phylogenetic analysis classified these isoforms into four evolutionary clades (Type I–IV), with *IbCHI1*, *IbCHI2*, and *IbCHI5* clustering within Type I/II subfamilies that retain conserved catalytic residues (e.g., Thr/Met 95, Ser/Thr 104, Gln 107) essential for enzymatic activity (Figure S1). Functional validation in transgenic callus lines confirmed that *IbCHI1* overexpression significantly enhanced total flavonoid content (1.36-fold, *p* < 0.01), while *IbCHI2* exhibited partial activity (1.39-fold, *p* < 0.05) (Figure 3b). In contrast, *IbCHI3* and *IbCHI4* showed no catalytic contribution, aligning with their classification into Type III/IV clades characterized by substitutions in critical substrate-binding residues (e.g., Lys 93 to Met, Tyr 108 to Val) (Figure S1). Subcellular location of IbCHI1, IbCHI2, IbCHI3 and IbCHI5 proteins were predicted to be cytoplasm. The flavonoid biosynthesis pathway is cytoplasmic, and the subcellular location in the cytoplasm of CHI gene family members has been proven in multiple species [34,35]. Chloroplast localization is unusual for CHI gene family members, but it has been reported in soybean [22], where the type III CHI members, GmCHI3A1 and GmCHI3A2 proteins were located to the chloroplast. In our results, *IbCHI4* (chloroplast localization) also belongs to type III CHI genes (sub-grouped with *AtFAP3*). The AtFAP3 protein was chloroplast localization and involved in chloroplast fatty-acid biosynthesis [10], thereby, *IbCHI4* was speculated to have similar functions to *AtFAP3*, associated with fatty acid metabolism rather than flavonoid synthesis [10], while *IbCHI3* (Type IV) may act as a non-catalytic enhancer of flavonoid production, as proposed in other species [32,33]. However, the absence of flavonoid accumulation in *IbCHI3*/*4*-overexpressing lines suggests species-specific functional divergence, possibly reflecting alternative roles in stress adaptation or metabolite transport.

All five *CHI* genes in sweet potato have typical conserved domains of the chalcone family, indicating their important role in regulating flavonoid synthesis (Figure 4). Gene replication plays an important role in the expansion of the genome, and many new gene functions are derived through gene replication, which is one of the main driving forces behind plant evolution [36,37]. The whole genome identification results showed that the five *CHI* genes of sweet potato were unevenly distributed on chromosomes 1, 5, 11, and 13 of sweet potato. The study found that there was collinearity between sweet potato and *CHI* genes such as *Arabidopsis*, *Ipomoea triloba*, and *Ipomoea trifida*, but there was no collinearity between sweet potato *IbCHI* genes. This indicates that the gene replication of sweet potato *CHI* gene family is different from that of *Arabidopsis*, *Ipomoea triloba*, *Ipomoea trifida*, etc. (Figure 5).

Non biological stresses such as salt, drought, and extreme temperatures can have negative effects on plant growth and development [38,39]. This study found that in addition to the basic elements, TATA box and CAAT box, shared by eukaryotes, the photoresponsive element Box 4, the methyl jasmonate (MeJA)-related response elements TGACG motif and CGTCA motif, the abscisic acid (ABA) response element ABRE, and stress responsive cis-acting elements such as drought-responsive element MBS and low temperature responsive element LER were also present in the promoter sequences of sweet potato *CHI* gene family members (Figure 4). Previous studies have shown that *CHI* genes play a role in plant abiotic stress, growth and development, and hormone signaling. As *CHI* catalyzes the conversion of chalcones into flavonoids, providing precursors for the biosynthesis of anthocyanins, the expression levels of *MmCHI1* and *MmCHI2* are positively correlated with anthocyanin content during the ripening process of mulberry fruit [40]. Under low-temperature treatment, the expression of six anthocyanin biosynthesis genes, including *CHI*, significantly increased in eggplant [41]. The expression of the *CHI* gene has tissue specificity. A study on the relationship between *CHI* transcription and anthocyanin staining in different parts of wheat plants found that the regulatory genes controlling anthocyanin staining in stems and peels regulate the transcription of *CHI* genes [42]. The expression level of the *IiCHI* gene in the aboveground part of Isatis indigotica is higher than that in the underground part. The flower has the highest expression level in the aboveground part, followed by the leaves and stems [43]. In Lonicera macranthoides, the *CHI* gene has the highest expression level in flowers, followed by stems and leaves [44]. This study analyzed the tissue-specific expression of sweet potato tissue using qRT-PCR. The results showed that the *IbCHI* gene in sweet potato had the highest expression level in the stem, with the expression levels of *IbCHI1*-*IbCHI4* being stem > root ≈ leaf, and *IbCHI5* being stem > leaf > root. Under abiotic stresses, *IbCHI* genes exhibited temporally phased and isoform-specific induction. During cold stress, *IbCHI1* and *IbCHI5* were rapidly activated (peaking at 3 h), coinciding with the early surge in total flavonoids (peaking at 12 h) (Figure 7b). This underscores their catalytic roles in initiating flavonoid production for cold adaptation, consistent with *IbCHI1*’s confirmed enzymatic activity (Figure 3b). In contrast, salt stress triggered synchronous upregulation of *IbCHI1*, *IbCHI4*, and *IbCHI5* at 3 h, with all isoforms peaking at 6 h alongside flavonoid accumulation (Figure 8b). Notably, *IbCHI4* (Type III/FPA3 subclass), despite lacking catalytic activity, displayed salt responsiveness, suggesting a non-enzymatic role in stress signaling or metabolite channeling, as proposed for Type III CHIs in fatty acid-associated pathways [10]. Drought stress elicited a distinct regulatory strategy; while upstream flavonoid genes were strongly induced, *IbCHI* genes showed transient, staggered activation. *IbCHI1* peaked earliest (3 h), correlating with the rapid flavonoid surge at this stage, whereas *IbCHI4* and *IbCHI5* peaked later (12 h) as flavonoid levels declined (Figure 9b). This temporal decoupling implies that *IbCHI1* drives the initial flavonoid synthesis under drought, while delayed *IbCHI4* and *IbCHI5* induction may mitigate oxidative damage or stabilize metabolic intermediates. The lack of catalytic activity in *IbCHI4* and *IbCHI5* (Figure 3b) further supports their auxiliary roles in stress adaptation, potentially through scaffold functions or interaction with other enzymes. The phased expression dynamics (early, middle, late stages) across stresses highlight a conserved regulatory logic: rapid activation of catalytic isoforms (*IbCHI1*, *IbCHI5*) during acute stress, followed by involvement of non-catalytic paralogs (*IbCHI3* and *IbCHI4*) in sustained adaptation. However, unlike *IbCHI1*’s consistent stress responsiveness, *IbCHI2* and *IbCHI3* showed minimal induction under all treatments, suggesting functional redundancy or roles in developmental rather than stress-related flavonoid synthesis.

This study identifies five chalcone isomerase (CHI) genes (*IbCHI1*–*IbCHI5*) in sweet potato, revealing their structural divergence, evolutionary classification into four clades (Type I–IV), and functional specialization. The catalytic CHI genes *IbCHI1* and *IbCHI5* serve as primary responders to abiotic stress, while non-catalytic members *IbCHI3* and *IbCHI4* may fine-tune metabolic flux or participate in stress signaling. This modular regulation, coupled with stem-dominated expression, positions sweet potato CHIs as key players in balancing growth and stress adaptation through flavonoid metabolism. Future research can further explore the transcriptional regulation mechanism of the *IbCHI* gene and attempt to improve its potential in improving crop quality and enhancing stress resistance through genetic transformation technology, thereby achieving precise optimization of sweet potato quality and yield.

## 4. Materials and Methods

### 4.1. Experimental Materials

The sweet potato variety ‘EC03′ was cultivated at the Food Crops Research Institute of the Hubei Academy of Agricultural Sciences. The healthy and uniform seedlings of ‘EC03′ were selected and maintained in clean water for one week. For stress treatment, the plants were exposed to a 4 °C condition for low-temperature stress, 200 mmol·L^−1^ NaCl solution for salt stress, and 20% polyethylene glycol 6000 (PEG-6000) for drought stress. Young leaves were collected at 0, 1, 3, 6, 12, and 24 h after treatment. The fibrous roots, stems, and leaves of sweet potatoes were collected from the same period (root and seedling stage) for tissue-specific expression analysis of subsequent *IbCHI* gene family members. All samples were immediately frozen in liquid nitrogen and stored at −80 °C after collection.

### 4.2. Total RNA Extraction and cDNA Synthesis

Total RNA was extracted from ‘EC03′ leaves using a plant RNA extraction kit (Vazyme Biotech Co., Ltd., Nanjing, China). The RNA integrity was verified via 1% agarose gel electrophoresis. High-quality total RNA was used for first-strand cDNA synthesis with a reverse transcription kit (TransGen Biotech Co., Ltd., Beijing, China). The reaction system contains total RNA 1 μg, 5× TransScript^®^ All in One SuperMix 4 μL, gDNA Remover 1 μL, and RNase-free water supplemented to a total volume of 20 μL. The reaction procedure includes incubation at 42 °C for 15 min and heating at 85 °C for 5 s. The obtained cDNA product was stored in a −20 °C freezer for further experimentation.

### 4.3. Identification of Sweet Potato CHI Gene Family Members

The sweet potato genome data and genetic structure annotations file were obtained from a sweet potato genome database (https://sweetpotao.com/) (accessed on 25 February 2025). The *AtCHI* were downloaded from the *Arabidopsis thaliana* database (https://www.arabidopsis.org/) (accessed on 25 February 2025). The candidate *CHI* gene family members were identified by BlastP comparison in sweet potato genome based on the query sequence of *AtCHI* genes. The Hidden Markov Model (HMM) of CHI (PF02431, PF16035, PF16036) was downloaded from the Pfam database (http://pfam.xfam.org/) (accessed on 25 February 2025) and compared in the sweet potato genome database by HMM 3.0 software with a threshold of E-value e^−5^. The *IbCHI* protein sequences were obtained by the overlap of the above two comparison methods with repeated sequence removal. SMART online tools (http://smart.embl-heidelberg.de/) (accessed on 25 February 2025) and NCBI—CDD (https://www.ncbi.nlm.nih.gov/Structure/cdd/wrpsb.cgi) (accessed on 25 February 2025) were used to identify the conserved domain of the *CHI* protein. The protein sequences without shared domain and complete domain were further removed.

### 4.4. Analysis of CHI Gene Family Members in Sweet Potato

The physicochemical parameters of the proteins, including amino acid length, molecular weight, isoelectric point, instability index, aliphatic index, and average hydrophilicity, were determined using the ExPASy ProtParam tool (https://web.expasy.org/protparam/) (accessed on 25 February 2025). Subcellular localization predictions were performed using the online platform CELL PLS (http://www.csbio.sjtu.edu.cn/bioinf/plant-multi/) (accessed on 25 February 2025). The secondary structure of IbCHI proteins was predicted through the SOPMA online server (https://npsa-prabi.ibcp.fr/cgi-bin/npsa_automat.pl?page=/NPSA/npsa_sopma.html) (accessed on 25 February 2025) while tertiary structures were modeled using SWISS-MODEL (http://swissmodel.expasy.org/) (accessed on 25 February 2025). Conserved motifs of CHI proteins were identified on the MEME website with parameters set to 8 motifs and a width range of 6–100 amino acids, and the results were visualized using TBtools software (Version v2.154) [45].

### 4.5. Evolution Analysis of Sweet Potato CHI Gene Family

The reported CHI genes from *Arabidopsis thaliana*, *Glycine max*, *Oryza sativa*, and *Solanum lycopersicum*, combined with 5 CHI genes from *Ipomoea batatas* were used for evolution analysis. In detail, the sequences were aligned by Cluster W in MEGA 7.0 software. The phylogenetic tree was constructed by the Neighbor-joining method with a Bootstrap value of 1500.

### 4.6. Collinearity Analysis of Sweet Potato CHI Gene Family

The chromosomal length and positional information of IbCHI genes were obtained from the sweet potato genome database. Intra- and interspecies collinearity analyses of IbCHI genes with homologs from *Arabidopsis thaliana*, *Ipomoea triloba*, and *Ipomoea trifida* were performed using TBtools [45]. Syntenic relationships between sweet potato and the three species were visualized to identify evolutionary conservation.

### 4.7. Chromosome Localization of Sweet Potato CHI Gene Family

The positional information of *CHI* gene family was extracted from sweet potato genome annotation files using TBtools software. A map of *CHI* gene family localization on sweet potato chromosomes was constructed.

### 4.8. Analysis of Cis-Acting Elements in the Promoter of Sweet Potato CHI Gene Family

The 2000 bp upstream promoter sequences of the CHI family genes were extracted from the sweet potato genome using TBtools software. Cis-acting elements within these sequences were analyzed through the PlantCARE online platform (https://bioinformatics.psb.ugent.be/webtools/plantcare/html/) (accessed on 25 February 2025). The results were streamlined and organized, followed by visualization using TBtools software.

### 4.9. Real-Time Fluorescence Quantitative PCR Analysis

Specific primers for the sweet potato CHI family genes were designed and synthesized by Ruike Biotechnology Co., Ltd. (Beijing, China) (Table S1), with β-actin selected as the internal reference gene. Quantitative real-time PCR (qRT-PCR) was conducted using Hieff^®^ QPCR SYBR Green Master Mix (Yisheng Biotechnology Co., Ltd., Shanghai, China) on a Bio-Rad CFX96 instrument, with triplicate reactions performed for each sample. The reaction mixture comprised 10 μL of Hieff^®^ QPCR SYBR Green Master Mix (No Rox), 0.5 μL each of forward and reverse primers, 4 μL of cDNA template, and 5 μL of RNase-free water. Thermal cycling conditions were set as follows: initial denaturation at 95 °C for 3 min, followed by 40 cycles of 95 °C for 10 s and 60 °C for 30 s. The relative expression levels of target genes were calculated using the 2^−ΔΔCt^ method [46].

### 4.10. The Construction of IbCHI-Overexpression Callus

The *IbCHI1*-*5* was cloned by PCR amplification from the cDNA of “EC03”. The fragments were detected in 1% agarose gel and recycled to connect to pMD 18-T vector. Then, the *IbCHI1*-*5* genes were inserted into pCAMBIA1300 overexpress vector with CaMV35S promoter [47] and transferred into *Agrobacterium tumefaciens* EHA105 (Sangon Biotech, Shanghai, China). Healthy leaf explants were surface-sterilized and cut into 5–8 mm disks under aseptic conditions. The disks were immersed in an Agrobacterium tumefaciens suspension (strain EHA105) harboring the *IbCHI*-overexpression vector for 15–20 min, followed by co-cultivation on MS medium (2 mg L^−1^ 2,4-D) supplemented with acetosyringone (100 μM) for 48 h in darkness. After rinsing to remove excess bacteria, the explants were transferred to a selection medium containing kanamycin (50 mg/L) and cefotaxime (250 mg/L) to induce callus formation.

### 4.11. Total Flavonoid Content Determination

The total flavonoid content was quantified using a spectrophotometric approach [48]. Fresh plant samples were homogenized in 80% ethanol (1:10, *w*/*v*) and centrifuged at 8000× *g* for 15 min. The supernatant was mixed with 5% NaNO_2_ (0.3 mL), 10% AlCl_3_ (0.3 mL), and 1 M NaOH (2 mL) sequentially, followed by incubation at 25 °C for 6 min. Absorbance was measured at 510 nm using a spectrophotometer (UV-1800, Shimadzu, Japan), with rutin as the standard (0–100 μg/mL). Total flavonoid content was calculated based on the standard curve. Triplicate measurements were performed for each sample.

### 4.12. Data Statistical Analysis

Statistical differences among groups were analyzed using a one-way analysis of variance (ANOVA) by SPSS 20.0. A significance threshold of *p* < 0.05 was applied to determine differences. The transcriptome analysis data comes from NCBI database with project identifier PRJNA486421 (Cold), PRJNA631585 (salt), and PRJNA413661 (drought). All experimental data were expressed as mean ± standard deviation (SD) of three biological replicates.

## Figures and Tables

**Table 1:** Physicochemical properties of *CHI* gene family in sweet potato.

Gene ID	Gene Name	CDS Size	Number of Amino Acids	Molecular Weight	Isoelectric Point	Instability Coefficient	Fat Coefficient	Average Hydrophilicity
*g3524*	*IbCHI1*	731	243	25,664.39	5.24	35.49	85.88	0.044
*g3586*	*IbCHI2*	815	271	28,730.63	4.89	41.19	88.86	−0.012
*g20441*	*IbCHI3*	740	246	27,319.24	4.78	38.89	96.22	0.005
*g54315*	*IbCHI4*	2381	793	88,752.07	9.22	38.68	93.2	−0.052
*g43784*	*IbCHI5*	1115	271	28,730.63	4.89	41.19	88.86	−0.012

**Table 2:** The secondary structure and subcellular location prediction of *IbCHI* protein.

Protein Name	α-Helix Proportion	Elongation Chain Proportion	β-Angle Proportion	Irregular Curl Proportion	Prediction of Cell Localization
*IbCHI1*	30.04	25.51	9.88	34.57	Cytoplasm
*IbCHI2*	27.68	24.72	8.86	38.75	Cytoplasm
*IbCHI3*	37.40	22.36	8.13	32.11	Cytoplasm
*IbCHI4*	38.21	18.41	8.07	35.31	Chloroplast
*IbCHI5*	27.22	25.88	5.93	40.97	Cytoplasm

## Data Availability

Data is contained within the article and Supplementary Materials.

## Supplementary Materials

The following supporting information can be downloaded at: https://www.mdpi.com/article/10.3390/plants14050752/s1, Figure S1: Alignment of IbCHIs with other CHIs. Blue circles over the residues indicate the conserved residues forming the substrate-binding cleft; Figure S2: The expression levels of *IbCHI1-5* in wild type (WT) and overexpression callus (OE1-3); Figure S3: The expression levels of *IbCHI1-5* in leaf, stem, and root; Table S1: Real-time fluorescent quantitative PCR primers for *CHI* gene family in sweet potato.
