# Supplementary material for: Bioinformatics and Expression Analysis of CHI Gene Family in Sweet Potato
Source: Plants (Basel). 2025 Mar 1;14(5):752. doi: 10.3390/plants14050752 (PMC11902207; doi:10.3390/plants14050752)
Supplement: Supplementary file 1 [file plants-14-00752-s001.zip › Supplementary Figures.pdf]

Supplementary Materials

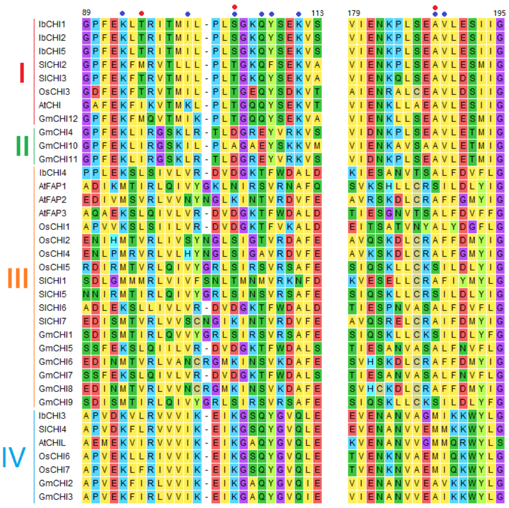

Figure S1. Alignment of IbCHIs with other CHIs. Blue circles over the residues indicate the conserved residues forming the substrate-binding cleft. Red circles indicate the residues participating in the hydrogen bond networks with substrates and catalytic water molecules.

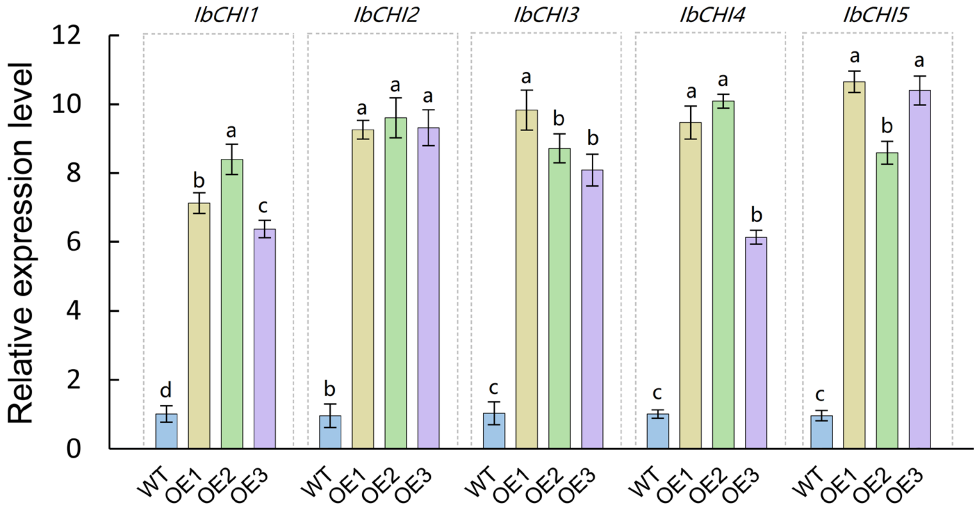

Figure S2. The expression levels of *IbCHI1-5* in wild type (WT) and overexpression callus (OE1-3). The expression levels in OE lines were 6.08–11.1-folds of WT. The lowercase letters indicate significant differences at $p < 0.05$ in samples within each gene.

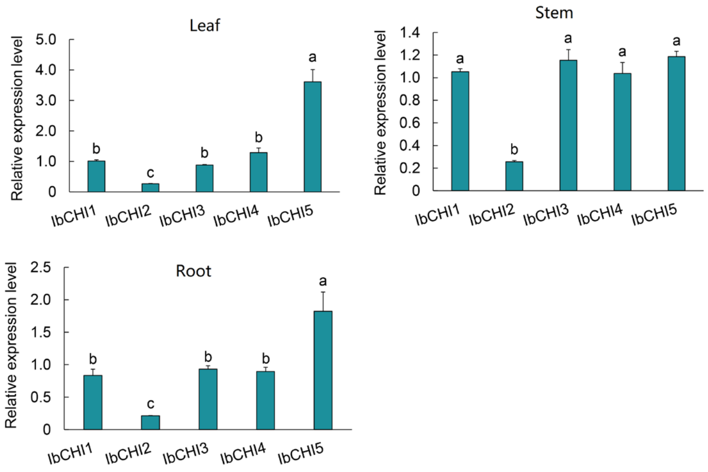

Figure S3. The expression levels of *IbCHI1-5* in leaf, stem, and root. The lowercase letters indicate significant differences at $p < 0.05$.
