# Supplementary material for: Bioinformatics and Expression Analysis of CHI Gene Family in Sweet Potato
Source: Plants (Basel). 2025 Mar 1;14(5):752. doi: 10.3390/plants14050752 (PMC11902207; doi:10.3390/plants14050752)
Supplement: Supplementary file 1 [file plants-14-00752-s001.zip › Table S1.pdf]

Table S1. Real-time fluorescent quantitative PCR primers for *CHI* gene family in sweet potato.

Primer name	Sequence (5′→3′)
g3524-F	GTGACCAAATGTTCCCACCG
g3524-R	CGACAATCTGACAGCGAGGC
g3586-F	GATGATACTTGTTGGCGGTGAC
g3586-R	CTTGCTGCCGATGATGGAC
g20441-F	TCAATCTGGACAATAAGCTGCTATC
g20441-R	TGATCTCCTTAATCACCACCACTC
g54315-F	TGCTCATAGCCGCCCTGA
g54315-R	TCTCCATGCCATAGCCTGTAAG
g43784-F	GCTGTATTAGTGCCACCGTTTG
g43784-R	CCAAGATGGCAGGAAACTCAA
β-Actin-F	AGCAGCATGAAGATTAAGGTTGTAGCAC
β-Actin-R	TGGAAAATTAGAAGCACTTCCTGTGAAC
